# Utilization of Sodium Nitroprusside as an Intestinal Permeation Enhancer for Lipophilic Drug Absorption Improvement in the Rat Proximal Intestine

**DOI:** 10.3390/molecules26216396

**Published:** 2021-10-22

**Authors:** Hisanao Kishimoto, Kaori Miyazaki, Hiroshi Tedzuka, Ryosuke Ozawa, Hanai Kobayashi, Yoshiyuki Shirasaka, Katsuhisa Inoue

**Affiliations:** 1 Department of Biopharmaceutics, School of Pharmacy, Tokyo University of Pharmacy and Life Sciences, Tokyo 192-0392, Japan; kisimoto@toyaku.ac.jp (H.K.); 0817km383@gmail.com (K.M.); serithruby@gmail.com (H.T.); ryo199408269005@gmail.com (R.O.); hanai.5884@gmail.com (H.K.); 2 Faculty of Pharmacy, Institute of Medical, Pharmaceutical and Health Sciences, Kanazawa University, Kanazawa 920-1192, Japan; shira@p.kanazawa-u.ac.jp

**Keywords:** sodium nitroprusside, absorption enhancer, mucus layer, passive diffusion, lipophilic drug

## Abstract

As advanced synthetic technology has enabled drug candidate development with complex structure, resulting in low solubility and membrane permeability, the strategies to improve poorly absorbed drug bioavailability have attracted the attention of pharmaceutical companies. It has been demonstrated that nitric oxide (NO), a vital signaling molecule that plays an important role in various physiological systems, affects intestinal drug absorption. However, NO and its oxidants are directly toxic to the gastrointestinal tract, thereby limiting their potential clinical application as absorption enhancers. In this study, we show that sodium nitroprusside (SNP), an FDA-approved vasodilator, enhances the intestinal absorption of lipophilic drugs in the proximal parts of the small intestine in rats. The SNP pretreatment of the rat gastrointestinal sacs significantly increased griseofulvin and flurbiprofen permeation in the duodenum and jejunum but not in the ileum and colon. These SNP-related enhancement effects were attenuated by the co-pretreatment with dithiothreitol or c-PTIO, an NO scavenger. The permeation-enhancing effects were not observed in the case of antipyrine, theophylline, and propranolol in the duodenum and jejunum. Furthermore, the SNP treatment significantly increased acidic glycoprotein release from the mucosal layers specifically in the duodenum and jejunum but not in the ileum and colon. These results suggest that SNP increases lipophilic drug membrane permeability specifically in the proximal region of the small intestine through disruption of the mucosal layer.

## 1. Introduction

The development of an improvement approach for the bioavailability of poorly absorbed drugs has been an important challenge in the field of oral drug delivery for decades. The progress in chemical synthesis has enabled the synthesis of molecules with higher molecular weight and complex structures. However, the field faces some problems related to gastrointestinal drug absorption due to the physicochemical properties of these molecules. The major problems are generally based on high lipophilicity and low solubility. To overcome these problems and to increase the oral bioavailability of these drugs, several types of absorption enhancers, including solvents, chelators, bile salts, surfactants, and polymers have been investigated [1,2]. These absorption enhancers are concomitantly administered with a lipophilic drug to increase its solubility in the gastrointestinal fluid, resulting in an accelerated passive diffusion across the mucosa. However, these effects are assumed to be nonspecific and cytotoxic, thereby limiting potential clinical applications.

Early studies have demonstrated that nitric oxide (NO), a gaseous signaling molecule involved in various physiological systems [3], modulates the intestinal permeation of hydrophilic compounds including that of macromolecules [4,5]. This mechanism involves the downregulation of intercellular tight junctions, regulating paracellular permeability in intestinal epithelia. However, our recent study revealed that NOC-7, spontaneously releasing NO, enhances not only hydrophilic but also lipophilic drug intestinal permeability [6]. Interestingly, these effects could be distinguished site specifically in the gastrointestinal tract. The permeation-enhancing effects on lipophilic and hydrophilic drug are specific to the proximal and the distal regions, respectively. The unique NOC-7 feature suggests its potential application in promoting oral lipophilic drug absorption. However, the safety of NO donors, such as NOC-7, has not been established in humans [7] and the chemical stability is rather low when dissolved (e.g., the NOC-7 half-life is approximately 5 min [8]).

Sodium nitroprusside (SNP), a metal-NO complex, is a traditional vasodilator approved by the U.S. Food and Drug Administration (FDA) and has already been established in clinical practice [9]. SNP is both chemically and physically stable under normal conditions [9,10,11] but releases NO in the presence of sulfhydryl groups [12]. Several studies have shown that SNP increases paracellular permeability in intestinal cell lines and the intestine of animal models, with is in good agreement with the role of NO releasers [13,14]. These findings imply that SNP might also be able to enhance lipophilic drug intestinal permeability as a stable and safe absorption enhancer.

In this study, we evaluated the effect of SNP on drug absorption in the rat small intestine. To investigate how SNP could affect intestinal drug absorption, we selected various compounds, known to be permeated mainly by passive diffusion, with different physicochemical properties, such as lipophilicity (log D). Furthermore, we explored whether SNP alters mucosal integrity, based on our recent finding that mucins regulate lipophilic compound permeation in the rat proximal intestine [15].

## 2. Results

### 2.1. SNP Pretreatment in the Rat Duodenum Increased Griseofulvin Transcellular Permeability

**Figure 1:**
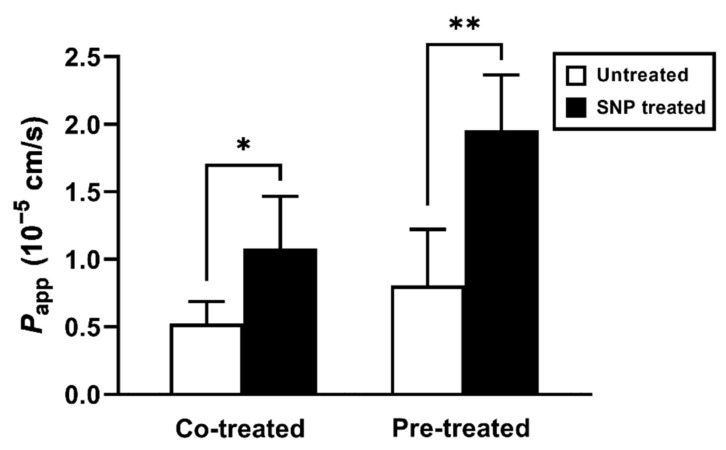
SNP effect on the apparent permeability coefficient (*P_app_*) of griseofulvin in the rat duodenum. The white and black bars show the untreated conditions as controls and the SNP-treated conditions, respectively. In the pre-treatment condition, the rat duodenums were treated with SNP (3 mM) for 60 min. The results are presented as the mean ± S.D. (*n* = 4–11) from 3 independent experiments. * *p* < 0.05, ** *p* < 0.01 compared with the control condition.

We previously demonstrated that exogenous NO enhanced the absorption of griseofulvin, a typical lipophilic drug, in the rat duodenum [6]. To examine whether SNP could increase intestinal absorption, we evaluated griseofulvin permeation in the rat duodenal sacs (Figure 1). Both co- or pre-treatment with SNP significantly increased griseofulvin permeation in the duodenum compared with the control. However, the enhancement effects of SNP differed between the co-and pre-treatment conditions. The pretreatment enhanced griseofulvin permeability more significantly compared with the co-treatment. These results were comparable with those of previous studies using NOC-7 as an NO releaser [6], suggesting that, similar to NOC-7, SNP can alter the transcellular permeation of lipophilic drugs in the rat duodenum.

**Figure 2:**
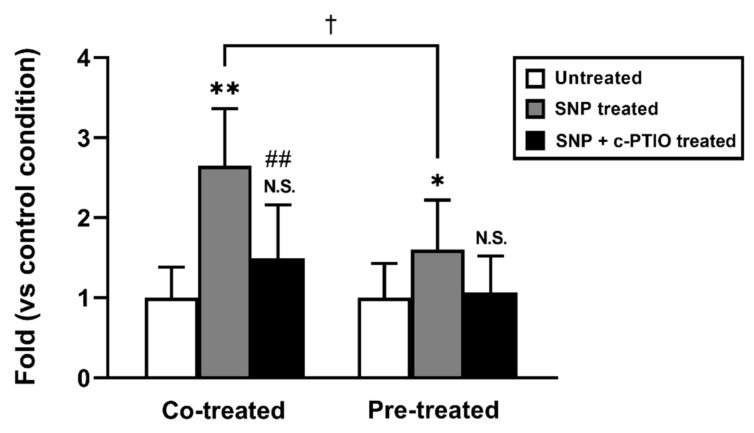
SNP effect on the FD-4 membrane permeation in the rat duodenum. The white, gray, and black bars show the untreated condition as a control, the SNP-treated conditions, and the SNP and c-PTIO-treated conditions, respectively. In the pre-treatment condition, the rat duodenums were treated with SNP (3 mM) with or without c-PTIO (1 mM) for 60 min. Results are presented as the mean ± S.D. (*n* = 4–12) from 3 independent experiments. * *p* < 0.05, ** *p* < 0.01 compared with the control condition. ^##^ *p* < 0.01 compared with the SNP-treated condition. ^†^ *p* < 0.05 compared with the co-treated condition. N.S., non-significant.

However, it has been demonstrated that NO opens tight junctions in the intestinal epithelial cells [4,5]. To rule out the involvement of the paracellular route in the SNP-enhancing effect in griseofulvin permeation, we evaluated the permeation of FD-4, a typical poorly absorbable hydrophilic compound that is commonly used as a paracellular marker, in the SNP-treated duodenum (Figure 2). Both the SNP co- and pre-treatments significantly increased FD-4 permeation. However, the pre-treatment effect was significantly lower than that of the co-treatment condition. These effects disappeared with c-PTIO, an NO scavenger, indicating that NO and NO-delivered molecules mediate the SNP effect.

When comparing the SNP effect on duodenal griseofulvin and FD-4 permeation, the former was higher while the latter was minimal under the SNP pretreatment conditions. These results indicate that SNP affects different targets between the transcellular and paracellular routes.

### 2.2. SNP Pretreatment in the Rat Intestine Showed Regional Differences in the Absorption Enhancement Effect

**Figure 3:**
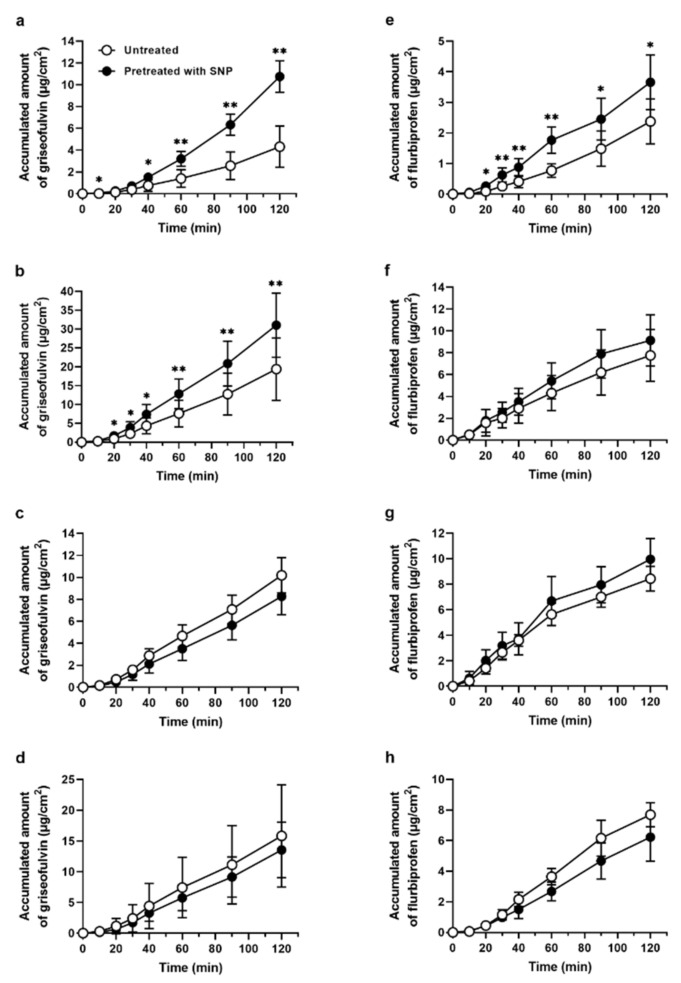
SNP effect on the intestinal griseofulvin and flurbiprofen permeation in rat gastrointestinal sacs prepared from the (**a**,**e**) duodenum, (**b**,**f**) jejunum, (**c**,**g**) ileum, and (**d**,**h**) colon. Time-course profiles of the accumulated amount in the serosal solution untreated (open circle) and pretreated with SNP 3 mM for 60 min (closed circle). The results are presented as the mean ± S.D. (*n* = 3–11) from three independent experiments. * *p* < 0.05, ** *p* < 0.01 compared with the control condition.

To examine the regional effect of SNP pretreatment in the rat small intestine, we determined the permeation of two different lipophilic drugs, griseofulvin and flurbiprofen, in the duodenum, jejunum, ileum, and colon. Figure 3 shows the results of the intestinal permeation studies with or without SNP pretreatment. Intestinal griseofulvin permeation significantly increased in the rat duodenum and jejunum upon the SNP pretreatment compared with that of the control (Figure 3a,b). However, the ileal and colonic permeation was not affected, suggesting a regional difference in the SNP effect (Figure 3c,d). In the case of flurbiprofen, a similar regional difference could be observed, as the intestinal permeation significantly increased in the rat duodenum but not in the jejunum, ileum, and colon (Figure 3e–h). We previously showed the pre-treatment of NOC-7 increased the absorption of griseofulvin in the rat duodenum [6]. Therefore, these results suggest that SNP might act like NOC-7 as a permeation enhancer to increase the transcellular permeation of lipophilic drugs in the proximal intestinal region.

### 2.3. SNP Showed a Mucus-Removing Effect in the Rat Intestine, Contributing to the Permeation Enhancement Effect

**Figure 4:**
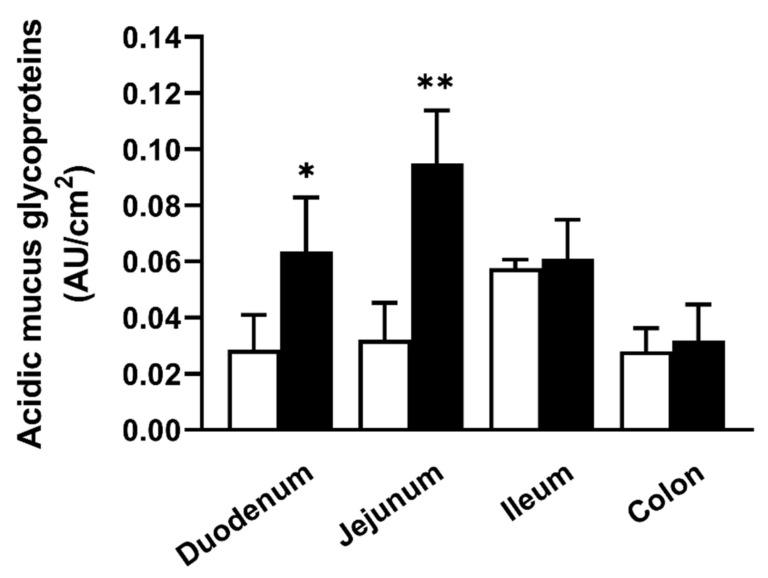
The amount of acidic glycoproteins released into the luminal solution in the rat intestinal sacs. The white and black bars show the untreated condition as a control and the pre-treated (for 60 min before the treatment with griseofulvin) conditions of SNP 3 mM, respectively. The results are presented as the mean ± S.D. (*n* = 3–6) from 3 independent experiments. * *p* < 0.05, ** *p* < 0.01 compared with the control condition.

Our recent finding that acidic mucins, such as Muc5ac, mainly contribute to the limitation of intestinal lipophilic drug absorption [15] could be implicated in the enhancing effect of NO and SNP in lipophilic drug permeation. To test the hypothesis that SNP disrupts the mucosal layers, thereby enhancing membrane permeation, we measured the amount of acidic glycoproteins released into the luminal solution in rat intestinal sacs (Figure 4). The acidic glycoprotein amounts in the SNP pre-treated samples significantly increased in the duodenum and jejunum compared with the untreated control conditions, while those in the ileum and colon did not change, suggesting that SNP has a mucus-removing effect exclusively in the proximal small intestine.

**Figure 5:**
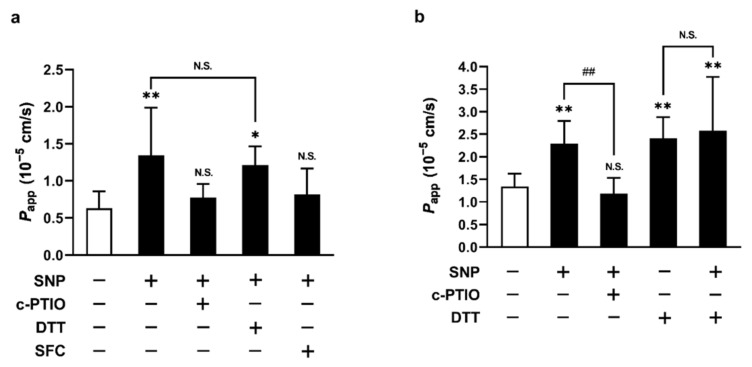
SNP, DTT, or SFC effects on the apparent permeability coefficients (*P_app_*) of griseofulvin (**a**) and flurbiprofen (**b**) in the rat duodenum. The white and black bars show the untreated condition as a control and the treated conditions; SNP (3 mM) pretreated for 60 min, c-PTIO (1 mM) treated with SNP, DTT (10 mM) treated for 10 min before the treatment with SNP, SFC (3 mM) pretreated for 60 min, respectively. The results are presented as the mean ± S.D. (*n* = 5–12) from 3 independent experiments. * *p* < 0.05, ** *p* < 0.01 compared with the control condition. ^##^ *p* < 0.01 compared with the SNP-treated condition. N.S., non-significant.

Based on our current data, we hypothesized that the permeation-enhancement effect of SNP could be explained by targeting the mucosal layer function. Therefore, we next investigated the possible SNP effector mechanisms. Figure 5 shows the results of the duodenal permeation studies of griseofulvin and flurbiprofen, examining the contribution of NO, the mucosal layers, or SNP structure on the permeation-SNP enhancement effect. Griseofulvin permeability was significantly increased by the SNP pretreatment and this enhancement effect was inhibited by the co-pre-treatment of SNP and c-PTIO (Figure 5a). However, the pretreatment of SFC, a structural SNP analog (without nitrosyl ligands), did not alter griseofulvin permeation. In addition, the SNP enhancement effect visibly disappeared when co-pretreated with SNP and DTT (Figure 5a). These results were more obvious in the case of flurbiprofen (Figure 5b). These results indicate the involvement of NO release and its potential targets as the mucosal layer.

### 2.4. Assessment of the SNP Pretreatment Effect on the Intestinal Permeability of Various Lipophilic Drugs

We evaluated the SNP pretreatment effect on the intestinal permeability of various lipophilic drugs. Table 1 shows the permeability (*P_app_*) of the tested drugs in different regions of the rat intestine, obtained under SNP-untreated and pre-treated conditions. We chose test compounds known to be permeated mainly by passive diffusion with different hydrophobicity (log D) under physiological conditions (pH 6.5), such as antipyrine, theophylline, and propranolol, including two tested drugs in the above-described study (griseofulvin and flurbiprofen). The SNP pretreatment effects in the proximal intestinal regions varied among the tested drugs, and their changing ratio was likely to be increased in the order of the log D values. This result suggests the possibility that the SNP effect depends on the log D values of test drugs.

## 3. Discussion

Although the NO-releasing chemical reagent enhances the absorption of lipophilic drugs in rats, NO and its oxidants are directly toxic to the gastrointestinal tract, thereby limiting their potential clinical application as absorption enhancers [7,16]. In this study, we demonstrated that SNP, a commonly used vasodilator drug for acute heart failure treatments, enhanced the site-specific intestinal permeation of lipophilic drugs in the rat small intestine by removing the mucosal layer. This finding suggests that SNP could be potentially used as an absorption enhancement reagent for lipophilic drugs administered via both the intestine and also various mucosal routes covered by mucosal layer, such as the nasal, ocular, and lung epithelia.

Our data show that SNP has a site-specific ability to increase the intestinal absorption of lipophilic drugs. Indeed, the SNP pretreatment enhanced the intestinal permeation of griseofulvin and flurbiprofen in the proximal region of the rat small intestine (Figure 1 and Figure 3). The log D (pH 6.5) values of griseofulvin and flurbiprofen were 2.17 and 1.50, respectively, suggesting that SNP affects the permeation of drugs with higher log D values. Although SNP reduces intracellular ATP levels that cause the tight junction barrier to dilate the paracellular route without cell damage [13,14], the contribution of the paracellular route in the SNP permeation-enhancing effect showed a modest FD-4 permeation increase upon SNP pretreatment (Figure 2). The results of this study using SNP are in good agreement with our previous findings showing that NOC-7 increases the duodenal permeation of lipophilic drugs [6]. Therefore, the mechanism involved in the permeation-enhancing effect by SNP is likely to be the same as NOC-7.

The site-specific SNP effect might be explained by its effect on the mucosal layers. In this study, we showed the mucus-removing effect of SNP, similar to DTT, suggesting the same SNP and DTT target (Figure 4). Furthermore, we confirmed an absence of combined effects and that the permeation-related SNP treatment effect apparently disappeared in the rat duodenum pretreated with DTT, although the DTT pretreatment increased the rat duodenal permeation of griseofulvin and flurbiprofen (Figure 5). Considering our findings, these data indicate that SNP alters the passive diffusion of lipophilic drugs in the rat duodenum by disrupting the structure of the mucosal layer. To the best of our knowledge, this is the first report on the mucus-removing effect of SNP pretreatment affecting the intestinal permeation of lipophilic drugs. We previously evaluated the effect of the mucosal layer on drug permeation in the rat small intestine using DTT, resulting in the involvement of acidic mucins, such as Muc5ac, in the limitation of intestinal lipophilic drug absorption [15]. Mucins are the main structural protein components of the mucosal layer [17,18,19], composing gel-like networks with viscoelastic properties and acting as dynamic barriers to limit intestinal lipophilic drug absorption [20,21]. DTT can remove mucus from the epithelium by breaking disulfide bonds, which compose the basic structure of mucus [15], whereas SNP is known to interact with sulfhydryl (SH) groups of certain proteins to produce NO [12]. Therefore, SNP acts as an NO donor and might also interact directly with extracellular proteins, such as mucin. Moreover, SNP might contribute to mucin dissociation. However, the underlying molecular mechanisms remain unclear. Recently, it has been reported that NO directly decreases the physicochemical properties of gel-forming mucins, such as viscosity and elasticity [22,23]. Although the mechanism underlying these reactions remains unclear [22], it is possible that the mechanism may be partially involved in the mucus-removing effect of SNP. Further studies would be required to clarify the mucosal layer-related SNP effector mechanism.

SNP is not active in oral administration; hence, it is clinically used in intravenous infusion [16]. Early clinical studies have demonstrated that oral SNP administration did not cause pharmacological effects, such as hypotension and hypothermia, in patients [9,16], suggesting low SNP bioavailability. This feature is suitable for an absorption enhancer. However, a high SNP dose and repeated administration cause SNP-derived cyanide accumulation, resulting in cyanide intoxication [9,16,24], although the clinical SNP dose has been proven to be safe. Therefore, further studies need to optimize the effect of SNP for use as a lipophilic drug absorption enhancer.

It has been reported that the exogenous NO induces cytotoxicity depending on the concentration of NO released from NO-releasing chemical reagents [25]. Since NO release from SNP is limited by the reaction with extracellular SH groups in certain proteins and reduced cysteine [12], SNP cytotoxicity is suggested to be lower than that of NO donors that rapidly release NO, such as NOC-7.

## 4. Materials and Methods

### 4.1. Materials

Sodium nitroprusside dihydrate (SNP) and fluorescein isothiocyanate-dextran 4,000 (FD-4) were obtained from Sigma-Aldrich Co. Ltd. (St. Louis, MO, USA). Carboxy-PTIO (c-PTIO) was obtained from Dojindo Laboratories (Kumamoto, Japan). Griseofulvin, antipyrine, theophylline, and sodium ferrocyanide decahydrate (SFC) were obtained from FUJIFILM Wako Pure Chemical Corporation (Osaka, Japan). Flurbiprofen was obtained from LKT Laboratories (St. Paul, MN, USA). Propranolol was obtained from Tokyo Chemical Industry Co., Ltd. (Tokyo, Japan). All other reagents were of analytical reagent grade.

### 4.2. Lipophilic Drug Intestinal Permeation Study

Animal experimental protocols (approval codes: P18-30, P19-09, and P20-14) were carried out in accordance with the Ethical Committee of Tokyo University of Pharmacy and Life Sciences. Male Wistar rats (8 weeks old) were obtained from Tokyo Laboratory Animals Science Co., Ltd. (Tokyo, Japan). Permeation studies of lipophilic drugs were carried out using the in vitro sac method, as previously described [6,15]. Briefly, rat gastrointestinal sacs were filled with 3 mL of Krebs–Henseleit bicarbonate buffer or 3 mM of SNP or SFC, an SNP structural analog, at the mucosal side and incubated at 37 °C for 1 h. Subsequently, the mucosal solution was replaced by 5 mL of drug solution containing 200 μM of each test compound (griseofulvin, flurbiprofen, antipyrine, theophylline, and propranolol) or 25 μM of FD-4, and the permeation study of the pretreatment condition was started. In the co-treatment condition, 3 mM of SNP was added to the drug solutions and the permeation study was started. To examine the effect of NO on permeation, 1 mM of carboxy-PTIO, an NO scavenger, was added to the SNP treatment. Furthermore, to examine the mucosal effect, 10 mM of DTT, a mucus remover, was supplemented as a pre-treatment for 10 min at the mucosal side before the SNP treatment. The serosal samples were collected at 0, 10, 20, 30, 40, 60, 90, and 120 min after administration, and analyzed using a reversed-phase HPLC system (LC-2000Plus; JASCO Co., Tokyo, Japan) or fluorescence microplate reader to determine the concentration of each test compound [15].

Based on these concentrations, the apparent permeability coefficients (*P_app_*) of each test compound were calculated according to the following Equation (1):

(1) $$P_{app}\left(\mathrm{cm}/s\right)=\frac{dQ}{dt}\times \frac{1}{C_{0}S}$$

where:

- *dQ/dt*—the flux of the test compound in the serosal side,
- *C*_0_—the initial concentration in the mucosal side,
- *S*—the surface area of the intestinal lumen.

### 4.3. Mucosal Glycoprotein Measurement

The amounts of mucosal glycoproteins were measured using alcian blue staining, as previously described [15]. Briefly, after incubation at the mucosal side with 3 mL of SNP solution (3 mM) for 60 min, the mucosal solutions with SNP were collected and concentrated for the solution volume of 150 μL by ultrafiltration using a 100-kDa molecular weight cut-off filter (Merck Millipore, Billerica, MA, USA) at 3000× *g* rpm for 30 min. Next, the solution was mixed with alcian blue (0.1% *w*/*v* alcian blue solution in 0.1 M sodium acetate buffer, pH 5.8, containing 25 mM MgCl_2_) at 4:1 and incubated (for 2 h at room temperature) to form mucus–dye complexes. Subsequently, these complexes were measured using a fluorescence microplate reader at an absorbance of 620 nm.

### 4.4. Data Analysis

All results are expressed as the mean ± standard deviation (mean ± S.D.). The statistical significance between the groups was analyzed using Student’s t-test or ANOVA followed by Dunnett’s method, and *p*-values of *p* < 0.05 were considered significant.

## 5. Conclusions

In this study, we demonstrated that SNP, a commonly used vasodilator drug, enhanced the site-specific intestinal permeation of lipophilic drugs via affecting diffusion through the transcellular route in the proximal region of the rat small intestine. Furthermore, we found that SNP has a mucus-removing effect, and this effect is involved in the SNP permeation enhancement effect. Our findings could help drug repositioning of SNP as a novel and safe permeation enhancer for improving drug absorption.

## Figures and Tables

**Table 1:** The permeability (*P_app_*) of various compounds with different hydrophobicity (log D) in rat small intestine pretreated with SNP (3 mM).

	Log D (pH 6.5) ^a^	Intestinal Region	*P_app_* (10^−5^ cm/s)		
			CTRL	SNP	Ratio
Griseofulvin	2.17	Duodenum	0.80 ± 0.42	1.96 ± 0.41 **	2.45
		Jejunum	4.22 ± 2.00	7.03 ± 1.92 **	1.67
		Ileum	2.43 ± 0.60	1.84 ± 0.44	0.76
		Colon	3.96 ± 2.14	3.17 ± 1.11	0.80
Flurbiprofen	1.50	Duodenum	0.58 ± 0.17	1.30 ± 0.27 **	2.24
		Jejunum	2.60 ± 1.19	3.27 ± 1.29	1.26
		Ileum	3.39 ± 1.13	3.98 ± 1.77	1.17
		Colon	2.83 ± 0.25	1.94 ± 0.37 **	0.69
Antipyrine	1.22	Duodenum	1.37 ± 0.64	1.22 ± 0.36	0.89
		Jejunum	1.96 ± 0.52	2.26 ± 0.45	1.15
		Ileum	1.79 ± 0.40	2.15 ± 0.58	1.20
		Colon	1.62 ± 0.38	1.99 ± 0.30	1.23
Theophylline	−0.08	Duodenum	2.34 ± 0.32	2.80 ± 1.23	1.20
		Jejunum	1.71 ± 1.14	1.67 ± 0.73	0.98
		Ileum	2.37 ± 0.55	2.47 ± 1.36	1.04
		Colon	2.07 ± 0.17	1.23 ± 0.25 **	0.59
Propranolol	−0.32	Duodenum	0.11 ± 0.06	0.13 ± 0.07	1.18
		Jejunum	0.47 ± 0.10	0.43 ± 0.18	0.91
		Ileum	0.37 ± 0.10	0.39 ± 0.10	1.05
		Colon	0.21 ± 0.11	0.35 ± 0.16 *	1.67

Results are presented as the mean ± S.D. (*n* = 3–15) from 3 independent experiments. * *p* < 0.05, ** *p* < 0.01 compared with the control condition. ^a^ Estimated values; from the public database of chemicalize.org by ChemAxon (https://chemicalize.com, accessed on 7 June 2019).

## Data Availability

The data presented in this study are available on request from the corresponding author.

## Sample Availability

Samples of the compounds are not available from the authors.
